# Ecstasy as a Potential Cause for Diffuse ST Elevation in a 27-Year-Old Healthy Male: A Case Report

**DOI:** 10.7759/cureus.85795

**Published:** 2025-06-11

**Authors:** Rabih Nasrallah, Amin Ossaily, Mazen Al Hammoud, Anwar Nader, Georges Ghanem

**Affiliations:** 1 Cardiology, Lebanese American University School of Medicine, Beirut, LBN; 2 Cardiology, Lebanese American University Medical Center, Beirut, LBN

**Keywords:** acute coronary syndrome, case report, coronary vasospasm, ecstasy, mdma

## Abstract

Ecstasy, also known as 3,4-methylenedioxymethamphetamine (MDMA), is widely used as a recreational drug known for its stimulating effects. While acute coronary syndrome and coronary vasospasm are frequently observed following cocaine and methamphetamine use, there have been few reported cases linking them to ecstasy use. We present a case of a 27-year-old, previously healthy, male presenting with oppressive retrosternal chest pain and diffuse ST-segment elevation, despite a normal troponin, several days after ecstasy use. His presentation followed by a normal coronary angiography is most consistent with an episode of coronary vasospasm. It is plausible that the consumption of MDMA contributed to this presentation. MDMA can lead to cardiovascular issues by increasing serotonin, dopamine, and noradrenaline levels, which may cause symptoms like coronary vasospasm. Although conclusive evidence linking MDMA to acute coronary syndrome is limited, cases of transient coronary vasospasm and thrombosis have been reported, suggesting a need for prompt evaluation and management of such conditions.

## Introduction

Amphetamine-type stimulants rank as the third most commonly used category of illicit drugs globally [1]. 3,4-methylenedioxymethamphetamine (MDMA), also known as ecstasy, is a popular amphetamine derivative widely used by young adults [2]. Although categorized as a controlled substance in the mid-1980s, MDMA has gained popularity for its stimulant effects, frequently seen at "rave" parties. Ecstasy is known to cause euphoria, increased energy, and sociability among users. Pharmacologically, several effects of this molecule have been well described in the literature, including anxiety, depression, and agitation that may persist for several days [3]. Cases of ecstasy-induced hyperthermia, hypertension, arrhythmias, rhabdomyolysis, hepatic and renal failure, and stroke have also been reported [4]. The use of MDMA has further been linked to instances of sudden death and cardiovascular collapse. However, only a few cases of myocardial infarction following MDMA use have been reported, particularly when compared with other stimulants such as cocaine or methamphetamine [2,4-8]. In this case report, presented in accordance with the CARE checklist and outlining a clear timeline, clinical findings, diagnostic workup, and interventions, we describe a young male patient who experienced typical chest pain and ST elevation on ECG four days after a single-use dose of ecstasy. This delayed onset of myocardial infarction following MDMA use is unusual and adds to the limited literature documenting such cardiovascular complications in the absence of polysubstance use or binge consumption.

## Case presentation

A 27-year-old healthy male presents to the emergency department (ED) after a 30-minute episode of localized oppressive retrosternal chest pain without radiation to either arms or jaw, associated with palpitations. The patient denied any other symptoms. On presentation, he was afebrile with a heart rate of 88 bpm, blood pressure of 140/100 mmHg, and oxygen saturation was within normal limits on room air. His physical examination was unremarkable. Ten minutes after the presentation, the patient mentioned a spontaneous reduction of his chest pain.

The patient denied any significant medical history, including any allergies, previous hospitalizations, or surgical procedures. However, he reports feeling flu-like symptoms two weeks prior to presentation, which resolved on their own. He also reports being a tobacco smoker but denies consuming alcohol. However, he states consuming weed and MDMA occasionally. His last pill of MDMA was on New Year’s Eve, four days prior to presentation. Importantly, he also mentions feeling nonspecific chest discomfort, pleuritic and non-oppressive, not lasting more than ten minutes and occasionally associated with generalized fatigability and weakness for the past six months. Furthermore, he denies taking any medications. He also denies any relevant family history, including premature cardiovascular disease. 

**Figure 1:**
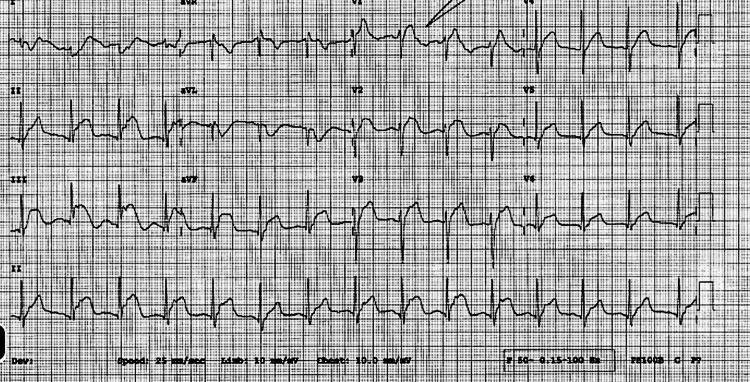
ECG recorded during chest pain at presentation. ECG tracing shows diffuse ST elevation coinciding with the patient’s chest pain upon presentation to the emergency department.

Electrocardiogram in the ED on presentation showed sinus rhythm with normal atrioventricular conduction and significant diffuse convex ST elevations most prominent in leads II, III, aVF, and V3-V6, without PR depression or reciprocal changes (Figure 1). Repeat ECG, ten minutes later, showed less pronounced ST changes relative to the index ECG, coinciding with improvement in the patient’s chest pain. The echocardiogram showed normal overall left ventricular systolic function with an ejection fraction of 60-65%. Diastolic function was also noted to be normal for age. Chambers were normal per size and volume criteria. There were no significant valvular structural or hemodynamic abnormalities, with no evidence of pulmonary hypertension. There were no signs of pericardial effusion. 

Laboratory workup showed a complete blood count within normal limits, despite neutrophilia (79.8%) and lymphopenia (11.5%). Serum electrolytes were within normal ranges. Creatinine levels were low at 0.62 mg/dL. Serum cardiac markers, including CK-MB (1 ng/dL; reference range <4.94 ng/dL) and troponin T (4 ng/L; reference range <15 ng/L), were both within normal limits. Repeated cardiac markers after three hours were within normal ranges. Urinalysis was also normal (Table 1). 

**Table 1:** Comprehensive laboratory report upon admission. This table summarizes the key laboratory findings of the patient. Overall, the complete blood count was within normal limits except for neutrophilia and lymphopenia. Serum electrolytes remained stable, while creatinine was noted to be slightly below the typical range. Cardiac markers including CKMB and troponin were within normal limits.

Parameter	Patient Value	Reference Range
WBC	6.5×10⁹/L	4.0-11.0×10⁹/L
Hemoglobin (Hb)	14.2 g/dL	13.5-17.5 g/dL (M)
RBC Count	5.0×10¹²/L	4.5-6.0×10¹²/L (M)
Hematocrit (Hct)	42%	41-53% (M)
Platelets	250×10⁹/L	150-400×10⁹/L
Neutrophils	79.8%	40-70%
Lymphocytes	11.5%	20-45%
CRP	2 mg/L	<5 mg/L
Sodium (Na⁺)	140 mmol/L	135-145 mmol/L
Potassium (K⁺)	4.2 mmol/L	3.5-5.0 mmol/L
Chloride (Cl⁻)	102 mmol/L	98-107 mmol/L
Bicarbonate (HCO₃⁻)	24 mmol/L	22-28 mmol/L
Calcium (Ca²⁺)	9.2 mg/dL	8.5-10.5 mg/dL
Magnesium (Mg²⁺)	1.9 mg/dL	1.7-2.2 mg/dL
Phosphate (PO₄³⁻)	3.5 mg/dL	2.5-4.5 mg/dL
Creatinine	0.62 mg/dL	0.7-1.3 mg/dL
Urea	5.0 mmol/L	2.5-7.1 mmol/L
CK-MB	1 ng/mL	<4.94 ng/mL
Troponin T	4 ng/L	<15 ng/L

The differential diagnosis included premature ST-elevation myocardial infarction (STEMI), type II myocardial infarction, coronary vasospasm, pericarditis, non-obstructive coronary arteries (MINOCA), and aortic dissection.

**Figure 2:**
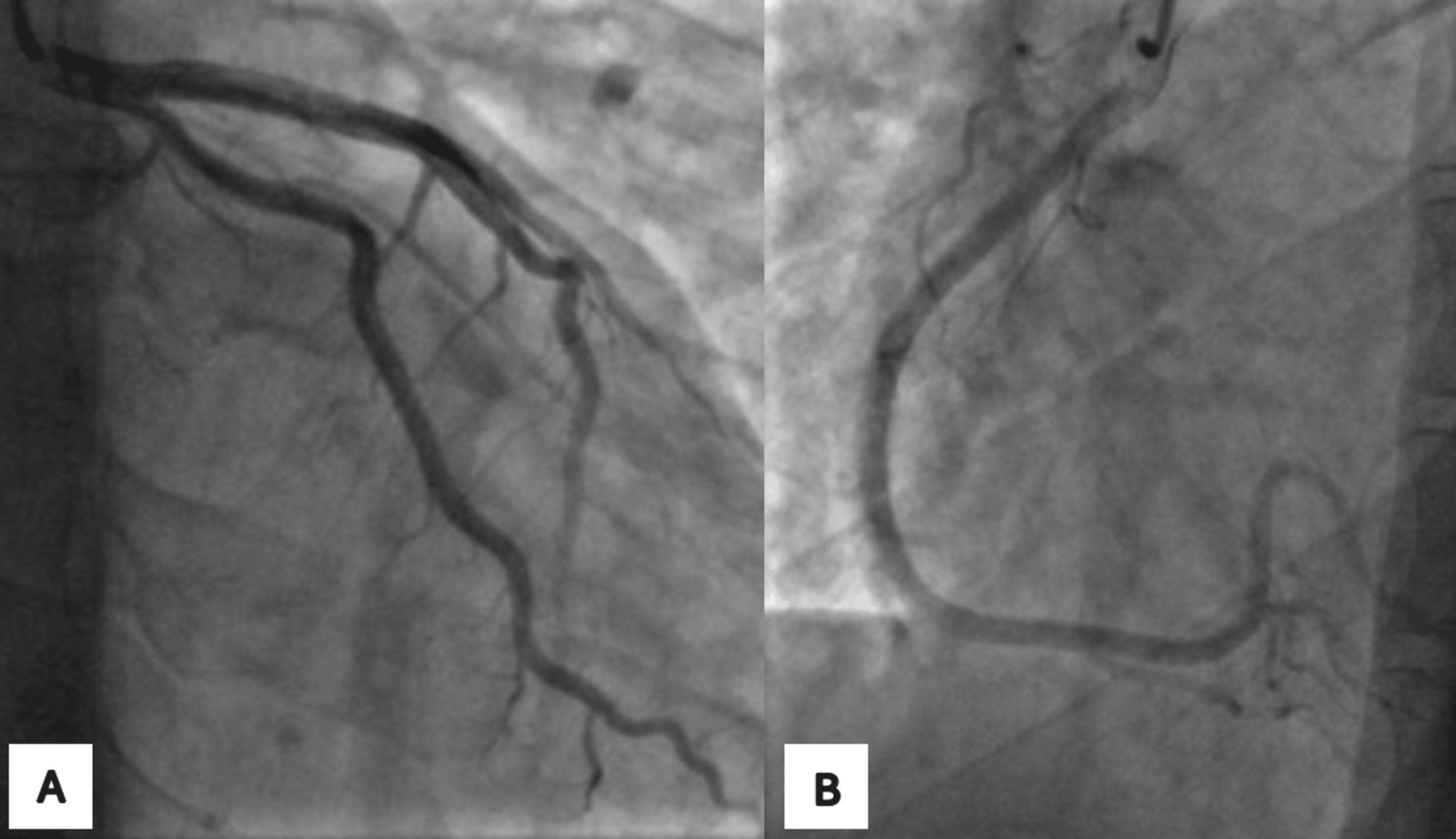
Coronary angiography of the left (A) and right (B) coronary arteries. Normal coronary angiography of the left (A) and right systems (B). No evidence of obstruction or flow disturbance.

**Figure 3:**
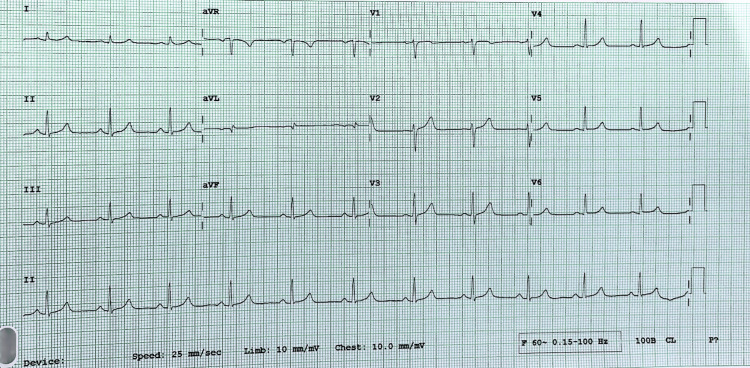
ECG prior to discharge after resolution of chest pain. ECG showing normal sinus rhythm with a resolution of the ST elevations seen upon arrival.

Left heart catheterization showed a normal left coronary tree (Figure 2). During the procedure, the engagement of the right coronary artery via a trans-radial approach with a 6-French catheter was difficult due to spastic arteries. The team used a 4-French catheter to investigate the right coronary artery and its branches, which turned out to be all normal. After catheterization, the patient's symptoms improved with the resolution of chest pain. His ECG prior to discharge was normal with total resolution of ST changes. (Figure 3). While provocative testing was not done, the patient presentation along with the transient ST elevation on ECG are most consistent with coronary vasospasm. 

Thus, the patient was then discharged home on the same day on Verapamil 80 mg daily. The patient was encouraged to pursue a rehabilitation program for smoking cessation and was advised to follow up in the cardiology clinic after one month to monitor his symptoms; however, he was lost to follow up. 

## Discussion

Although considered a safe drug, MDMA has been reported to cause detrimental side effects on several body organs, particularly the cardiovascular system. The pathophysiology of its toxicity is complex and not well understood. Several studies explain how MDMA causes an increase in the release of serotonin, dopamine, and noradrenaline not only in the central nervous system but also in peripheral tissues. MDMA blocks the reuptake transporters at different synapses, particularly those involved in serotonin reuptake, resulting in a rapid rise in the concentration of these neurotransmitters within the synapse [9]. The acute rise in noradrenaline primarily accounts for the drug's impact on the cardiovascular system, while high levels of serotonin are associated with peripheral vasoconstriction [10]. Typically, the effects of MDMA begin approximately 20 to 30 minutes after ingestion, lasting up to 48 hours, and may be intensified when combined with alcohol [5].

Little data is available about the relationship between ecstasy use and acute coronary syndrome or coronary vasospasm. Few cases have been reported in the literature within the past two decades [2,4-8]. Therefore, no definitive conclusions can be made regarding the underlying mechanism. Nevertheless, since ecstasy is a sympathomimetic drug similar to cocaine and methamphetamine, it is suggested that a similar mechanism, such as coronary vasospasm, may have contributed to the observed effects [11]. Acute coronary syndrome typically occurs within the first few hours of cocaine or methamphetamine use [12]. The delayed onset and prolonged duration of symptoms observed in our case following reported ecstasy use may not solely be explained by vasospasm. It has been reported in the literature that acute coronary thrombosis due to MDMA use may explain the delay in symptoms [2,11].

In our case, the patient presented with typical chest pain and ST elevations on ECG. Cardiac enzymes, including high-sensitivity troponin T and CKMB, were negative upon admission due to the patient's early presentation and remained normal on repeat sampling three hours later, supporting the absence of myocardial injury. Coronary angiography was performed and did not show any signs of thrombosis or spasm. Provocative vasospasm testing was not performed due to institutional policy and unavailability, which is a diagnostic limitation. The timing of the procedure coincided with the resolution of the patient’s symptoms, suggesting that the patient had an episode of coronary vasospasm that resolved before angiography. No signs of thrombosis were seen on angiography; however, the presence of small thrombi that may have dissolved spontaneously or embolized distally cannot be ruled out. This case may fall under the spectrum of myocardial infarction with non-obstructive coronary arteries (MINOCA), although vasospasm remains the leading hypothesis.

The patient denied intravenous drug use, effectively ruling out the possibility of septic emboli or endocarditis. This was further supported by the absence of clinical signs and the lack of any echocardiographic features suggestive of endocarditis. Unfortunately, a urine drug screen was not performed at the presentation due to logistical constraints and this limitation is acknowledged.

In this case, we opted for invasive coronary angiography due to the urgency and severity of the patient's symptoms, including rapid symptom resolution and diffuse ST-segment elevation. These factors raised concerns about significant coronary pathology that required immediate and definitive evaluation. However, it is important to consider that noninvasive coronary CT angiography (CTA) is also a valuable diagnostic tool, particularly in cases where the clinical risk is low to intermediate [13].

The patient was diagnosed with transient coronary vasospasm due to ecstasy use and was prescribed a calcium channel blocker (verapamil) to prevent further vasospastic episodes. Since there was no evidence of myocardial infarction or thrombosis, antiplatelet or anticoagulant therapy was not necessary. A significant challenge in diagnosing this patient was the inability to confirm the occurrence of vasospasm, as angiography results were normal. Additionally, the institution had ceased performing spasm provocation tests due to safety concerns and lacked other advanced imaging techniques. Such tests could have offered a definitive diagnosis and potentially clarified the patient’s condition.

In many instances, determining the precise type and quantity of recreational drugs can be challenging when patients require urgent treatment. While a standard dose ranges from one to two tablets, the MDMA content per tablet can vary significantly, sometimes by up to 100-fold, and these pills may also include other harmful substances [14]. The most reliable strategy for managing patients experiencing chest pain and ST-segment elevation following the use of sympathomimetic recreational drugs involves employing standard treatments for acute myocardial infarction while maintaining a relatively low threshold for performing coronary angiography and keeping the differential diagnosis of coronary vasospasm high on the list.

## Conclusions

This case report sheds light on the possible cardiovascular side effects of ecstasy use. While the exact role of MDMA in this patient's presentation remains uncertain, it highlights the importance of considering recreational drug use in the differential diagnosis of chest pain and ECG changes in young adults. Young adults presenting with typical chest pain and ECG changes following recreational drug use should undergo a complete workup to rule out acute coronary syndrome, including echocardiographic evaluation and coronary angiography. Coronary vasospasm should be considered a key differential diagnosis, even if symptoms occur later than the expected time frame.
